# The Continuing Evolution of Insulin-like Growth Factor Signaling

**DOI:** 10.12688/f1000research.22198.1

**Published:** 2020-03-23

**Authors:** Steven A Rosenzweig

**Affiliations:** 1 Department of Cell and Molecular Pharmacology & Experimental Therapeutics, Medical University of South Carolina, Charleston, SC, 29425, USA

**Keywords:** insulin-like growth factor1, signaling, cancer, Alzheimer's disease, dementia, type 2 diabetes, insulin resistance

## Abstract

The insulin-like growth factors (IGFs; IGF1/IGF2), known for their regulation of cell and organismal growth and development, are evolutionarily conserved ligands with equivalent peptides present in flies ( *D. melanogaster*), worms ( *C. elegans*) among others. Two receptor tyrosine kinases, the IGF1 receptor and the insulin receptor mediate the actions of these ligands with a family of IGF binding proteins serving as selective inhibitors of IGF1/2. This treatise reviews recent findings on IGF signaling in cancer biology and central nervous system function. This includes overexpression of IGF1 receptors in enhancing tumorigenesis, acquired resistance and contributions to metastasis in multiple cancer types. There is accumulating evidence that insulin resistance, a hallmark of type 2 diabetes, occurs in the central nervous system, independent of systemic insulin resistance and characterized by reduced insulin and IGF1 receptor signaling, and may contribute to dementias including Alzheimer’s Disease and cognitive impairment. Controversy over the role(s) of IGF signaling in cancer and whether its inhibition would be of benefit, still persist and extend to IGF1’s role in longevity and central nervous system function.

## Introduction

### IGF-1 and members of the IGF family

In mammals, the insulin-like growth factor (IGF) family comprises three ligands—IGF-1, IGF-2, and insulin—and three receptors—the IGF-1 receptor (IGF1R), insulin receptor (IR), and IGF-2 receptor (IGF2R). The IGF1R and IR are structurally related heterotetrameric receptor tyrosine kinases (RTKs), with the IR having two alternatively spliced isoforms, IRA and IRB, whose functions are incompletely understood ^1, 2^. The IR isoforms were recently shown to be regulated by their IGF-binding affinities and principally that of IGF-2 rather than their insulin-binding affinities; IGF-2 binds to IRA and IGF1R and is a more potent mitogen than IGF-1 ^3^. IRB modulates metabolic actions in adults while IRA functions in prenatal growth and development and mediates the mitogenic effects of insulin ^4^. IRA homodimers and IRA/IGF1R heterodimers have mitogenic actions and stimulate cancer cell growth ^5^. While beyond the scope of this review, it is noteworthy that considerable progress has been made in defining the 3D structure of the IGF1R and delineating how IGF-1 binds to and activates the receptor ^6– 9^. The IGF2R, also known as the mannose-6-phospate (M6P) receptor, is acknowledged for targeting newly synthesized lysosomal hydrolases from the endoplasmic reticulum to lysosomes. The IGF2R lacks a signaling function and instead binds to IGF-2 at the cell surface and clears it via receptor-mediated endocytosis and lysosomal degradation. *IGF2* is an imprinted gene expressed only from the paternal allele ^10, 11^.

In addition to the ligands and receptors, there is a family of six IGF-binding proteins (IGFBPs) exhibiting higher affinities (pM) for the IGFs than their cognate receptors (nM) that serve to tightly regulate IGF-1 and IGF-2 levels/bioavailability in the circulation and at the cell surface ^12^. Importantly, the IGFBPs lack any measurable affinities or selectivity for insulin, raising the following question: why are there six IGFBPs ^13^ and no insulin BPs? Consistent with these proteins’ evolutionary conservation, in addition to ligands and receptors, a recent report described the presence of an ancestral IGF-1/insulin-binding protein ^14, 15^. While there is no agreed-upon or absolute answer to this conundrum, a key piece of this puzzle relates to the sites and manner of insulin biosynthesis compared to that of the IGFs. Insulin biosynthesis occurs in the beta cells of the pancreatic islets of Langerhans, where it is stored in granules until it is needed in response to elevated glucose levels. In contrast, IGF-1 and IGF-2 are made by a number of cells where they undergo constitutive secretion rather than being stored in their cells of origin and serve as paracrine/autocrine factors. IGF-1 and IGF-2 are also synthesized and released into the circulation by the liver, where they exist free, in binary complexes with the IGFBPs or in ternary complexes of ~140 kDa with IGFBP3 or IGFBP5 plus an acid labile subunit prolonging its circulating half-life from hours to days following its secretion ^16^. IGF-1 production is under the control of pituitary growth hormone after birth ^11^.

It is noteworthy that in humans there is a single insulin, IGF-1, and IGF-2, whereas *Drosophila* has eight *Drosophila* insulin-like peptides (DILP-1–8) and *Caenorhabditis elegans* has upwards of 40 ILPs (reviewed in 14). In *Drosophila*, insulin-like polypeptide-binding protein (IBP) serves as an IGFBP homolog that was shown to have nM binding affinity with the following order of affinity: DILP-5 (K _d_ 2 nM) > IGF-1 (K _d_ 13.6 nM) > insulin (K _d_ 135 nM) ^14^. Crystallographic studies on *Drosophila* imaginal morphogenesis protein-late 2 protein (Imp-L2) bound to DILP-5 and bound to IGF-1 revealed that the overall structure of lmp-L2 differs significantly from that of the IGFBPs, comprising two immunoglobulin-like fold domains ^14^. To obtain more-detailed information on the contact sites between Imp-L2 and IGF-1, Pompach and co-workers used IGF-1 or des(63–70)-IGF-1, which lacks the C-terminal octapeptide and the loss of two out of three lysyl residues that were derivatized on their amino groups (N-terminal and lysyl residues), with a heterobifunctional cross-linker in order to photocrosslink complexes of Imp-L2:IGF-1 in a manner that went beyond previous photoaffinity labeling studies of IGFBP2 ^17^, enabling the co-identification of where different IGF-1 domains contact Imp-L2 ^15^. It was suggested that the regulation of ILP bioavailability is likely represented by an alternative strategy to the IGFBPs given their structural differences to the IGFBPs; because of the highly conserved nature of the IBPs in insects, this system may be exploited as a future therapeutic target for blocking the transmission of insect-borne diseases such as malaria ^14^. Of note, the ILPs signal through a single insulin receptor-like receptor ^18^, with DILP-8 signaling through a G-protein-coupled receptor, Lgr3 ^19^. This differs from humans, in whom signaling is mediated by the IGF1R, IGF2R, IRA, IRB, and associated hybrid heterotetramers. This suggests that ligand diversity in insects preceded receptor diversity, with greater ligand regulation occurring through the IBPs. The role of IGF-1 signaling in cancer and Alzheimer’s disease (AD) will be the focus of the remainder of this treatise.

## IGF-1 and cancer

IGF-1 signaling plays a role in cancer tumorigenesis and metastasis, which led to the IGF1R becoming a therapeutic target for multiple cancer sites ^20, 21^. Accordingly, a number of small-molecule RTK inhibitors (RTKIs) and monoclonal antibodies (mAbs) targeting the IGF-binding domain on the IGF1R were developed for use in treating cancer. To date, all of these strategies have failed in clinical trials, primarily owing to the onset of acquired resistance ^22, 23^. In reviewing anti-IGF therapeutics in breast cancer, Yee suggested that failure of the first generation of IGF1R inhibitors has unfairly reduced confidence in their use and that, as we learn from these mistakes, the death knell for these inhibitors may have been premature ^24^. Instead, he advocates that by identifying and then applying predictive biomarkers, a cohort of patients with IGF1R-driven tumors who will be more likely to respond positively to treatment may be identified; this approach has exhibited success in analyzing patients with lung cancer ^25^. It is notable that the IGF1R-targeting mAb teprotumumab was tested for safety and efficacy in a multicenter, double-masked, randomized, placebo-controlled trial in patients with active, moderate-to-severe ophthalmopathy associated with Graves’ disease ^26^. Teprotumumab (Tepezza®) was approved for use in January 2020 and is the first drug for use in adults for treating thyroid eye disease.

Over the years, serum levels of IGF-1, IGF-2, and IGFBP3 have been evaluated as predictive biomarkers for breast and prostate cancers, with elevated IGF-1 levels being assigned as a risk factor for prostate cancer ^27^. A review of the population-based epidemiologic studies reaffirmed the association of high serum IGF-1 levels with breast, prostate, colorectal, and other cancers ^28^. While these results remain controversial, it was recently reported that either low levels of IGFBP1 or elevated levels of IGF-1 increase the risk for prostate cancer ^29^. In that context, transgenic mice overexpressing IGF-1 in prostate epithelial cells exhibited spontaneous formation of prostate adenocarcinomas ^30^. To more directly address the question of whether elevated circulating IGF-1 levels contribute to prostate cancer, Wang and colleagues crossed transgenic mice overexpressing hepatic IGF-1 (HIT mice) with mice overexpressing human c-Myc in the prostate driven by the androgen-responsive probasin (ARR2Pb) promoter (Hi-Myc). These studies revealed that elevated serum IGF-1 in the Hi-Myc/HIT mice was associated with prostate enlargement, invasive prostate cancer, and more-aggressive prostate adenocarcinoma, which did not occur in Hi-Myc mice with normal levels of serum IGF-1 ^31^. In *C. elegans,* elevated insulin/IGF-1 led to accumulation of the human forkhead box O3A gene ( *FOXO3A*), a transcription factor/ *daf-16* homolog in *C. elegans* and downstream target of insulin/IGF-1 signaling that regulates lifespan and metabolism in lower organisms ^32^. FOXO3A expression is also associated with longevity in humans based on greater expression of FOXO3A polymorphisms (single nucleotide polymorphisms) in centenarians and nonagenarians compared to younger controls, suggesting that *FOXO3A* may be a susceptibility gene for prolonged survival in humans ^33^. *FOXO3A* is downstream of IGF1R signaling and activation of phosphoinositide 3-kinase (PI3K), phosphoinositide-dependent kinase 1 (PDK1), and Ak strain transforming (Akt; also known as protein kinase B) ^34^ and regulates the activation/inhibition of multiple target genes. In this pro-survival signaling pathway, activated Akt enters the nucleus and phosphorylates FOXO3A, downregulating Bcl-2-interacting mediator of cell death (Bim) to inhibit apoptosis and promote cell proliferation.

Pan and co-workers demonstrated that primary (not acquired) resistance to the epidermal growth factor receptor (EGFR) TKI gefitinib ^22^ in non-small cell lung cancer (NSCLC) cell lines was due, in part, to IGF1R signaling ^35^. They further showed that combining gefitinib with the oral hypoglycemic drug metformin restored gefitinib sensitivity to resistant NSCLC (H1975) cells, resulting in decreased phospho-Akt (pAkt) levels and elevated Bim expression ^35^. In this regard, Qiu and co-workers ^36^ reported that both primary and acquired resistance of breast cancer cell lines to the anti-human epidermal growth factor receptor 2 (HER2) mAb trastuzumab are both increased by Cullin7 (CUL7), a scaffold protein for the CUL7 E3 ubiquitin ligase that is overexpressed in the trastuzumab-resistant cells. The principal target of the CUL7 E3 ligase is Ser-phosphorylated insulin receptor substrate 1 (IRS1), a form of IRS1 that lacks the ability to signal through the PI3K/Akt pathway because of its reduced ability to be tyrosine (Tyr)-phosphorylated ^37^. Accordingly, CUL7 overexpression favors signaling through Tyr-phosphorylated IRS1, which is reinforced by CUL7 degradation of IGFBP3, in turn raising IGF-1 levels and IGF1R signaling, which then enhances resistance to trastuzumab ^36^. Related to these findings, Yang and co-workers reported that the tyrphostin NT157 downregulates IRS1/2 proteins in breast cancer cell lines by binding to the IGF1R, increasing serine phosphorylation of IRS1/2, leading to their degradation and reduced IGF1R signaling ^38^. Both of these studies provide support for targeting IRS protein degradation in NSCLC and breast cancer. While the mechanism of IRS degradation has yet to be defined ^38^, Qiu *et al*. ^36^ suggest that CUL7 levels may serve as a biomarker for trastuzumab responsiveness and combination therapy employing CUL7 deletion and trastuzumab.

## The brain IGF system: neurodegenerative disease and cognition

In addition to its well-known hormonal function regulating growth and influencing fuel metabolism and lifespan, the IGF system (including insulin signaling) has received considerable attention in recent years for its role in the central nervous system (CNS). Unlike insulin, which is exclusively synthesized by the beta cells of the pancreatic islets of Langerhans, IGF-1 is synthesized within various brain regions by neurons, glial cells, and endothelial cells, with systemic IGF-1 also accessing the brain by crossing the blood–brain barrier (BBB) ^39^. The role played by insulin and the IGFs in the CNS has been linked to brain development, metabolism, injury repair, cognition, and mood ^40^. Consequently, loss of insulin/IGF-1 signaling in the brain, mediated by both the IR and the IGF1R, has generally been associated with an elevated risk of AD, cognitive decline, (premature) dementia, depression, and anxiety ^41– 43^. IGF-1 signaling plays multiple roles in the CNS, and its potential beneficial role in AD and other dementias helps make the case for the IGF system as a therapeutic target in neurodegenerative diseases, including AD ^16^. In this context, IGF-1 functions both as a hormone following access of systemically produced IGF-1 to the CNS and as a locally produced autocrine/paracrine factor whose levels are tightly regulated by CNS-produced IGFBPs ^12^.

Mice with reduced levels of circulating IGF-1 (resulting from targeted disruption of hepatic IGF-1 expression) exhibit cognitive deficiencies based on impaired performance in a hippocampal-dependent spatial-recognition task and disrupted long-term potentiation (LTP) ^44^. These deficits are reversed by systemic administration of IGF-1, which normalized glutamatergic boutons in the hippocampus that were reduced in mice with low serum IGF-1 ^44^. In testing the impact of loss of insulin/IGF-1 signaling in the brain, Soto and co-workers ^45^ generated double knockout (DKO) mice with inactivated IR/IGF1R in the hippocampus (Hippo-DKO) or the central amygdala (CeA-DKO). These studies confirmed that spatial learning and memory require insulin/IGF-1 signaling in the hippocampus while insulin/IGF-1 signaling in the central amygdala mediates thermogenesis ^45^. Based on 7 Tesla MRI brain imaging of human subjects, elevating peripheral IGF-1 was positively linked to increased hippocampal volume and memory (verbal recall) ^46^. An opposing role for IGF-1 in the CNS was the subject of a recent review underscoring that in AD, reducing IGF-1 signaling may serve to clear protein plaques and slow disease progression while at the same time reduced IGF-1 levels may lead to dysregulated cognition and neurovascular function ^47^. Based on IGF-1’s ability to slow or reverse cognitive decline, quantifying its levels in the circulation has been promoted as a biomarker for cognitive decline ^48^. Further support for IGF-1 as a neurotrophic factor comes from work on Parkinson’s disease where low serum IGF-1 levels correlated with poor performance on executive tasks in early, drug-naïve patients and may be predictive of poor performance on attention/executive and verbal memory tasks after a 2-year follow-up ^49^. Thus, the role of systemic IGF-1 on neurogenesis, cognition, and dementia deserves further analysis.

## Type 2 diabetes mellitus, insulin/IGF-1 signaling, and AD

Controversy concerning the positive versus negative role(s) of insulin/IGF-1 signaling in the brain exists. For example, Kleinridders and co-workers reported that insulin signaling via brain IR/IGF1R alters systemic metabolism and a variety of brain functions ranging from appetite control and body temperature to neuronal function and synaptogenesis, providing evidence for a favorable role ^50^. Contradictory findings with respect to the role of insulin/IGF-1 signaling in neurodegenerative disease exist. Specifically, in humans, insulin resistance and type 2 diabetes mellitus (T2DM) are associated with AD. Moreover, patients with diabetes were at higher risk for dementia and patients with diabetes and dementia are at greater risk for severe hypoglycemia ^51^. In lower organisms, including *Drosophila*, *C. elegans*, and mice, reduced levels of insulin/IGF-1 signaling slows neurodegeneration and increases longevity (reviewed in 52). A similar inverse relationship between longevity and IGF1R levels was reported for 16 distinct rodent species ^53^; it did not extend to peripheral tissues or to body mass (which has been shown to be directly related to IGF-1 levels in purebred dogs ^54^). This led the authors to suggest that IGF1R signaling in nervous tissue played a role in the evolution of longevity in mammals ^53^. The expression levels of both the IR and the IGF1R are higher in younger mice and decrease as the animals age ^55^. As detailed above, insulin/IGF-1 signaling in the CNS is frequently quantified by IRS1/IRS2 phosphorylation, with elevated IRS1/2 serine phosphorylation contributing to AD. The assertion that decreased insulin/IGF-1 signaling contributes to reduced cognitive function supports the therapeutic approach of insulin or IGF-1 treatment in order to enhance cognition. However, contrary observations have been reported in animal models of AD where reduced insulin/IGF-1 signaling through IR/IGF1R and IRS2 slowed the progression of neurodegeneration, suggesting IRS2 action is a negative regulator of cognition/dementia ^56^. Decreased insulin/IGF-1 signaling is known to increase longevity in *C. elegans* ^57^, *Drosophila* ^58^, and transgenic mice ^59^ while lowering the incidence of multiple cancers by reducing tumor growth and metastasis ^60^. It is clear that the role of insulin/IGF-1 signaling in aging (beyond examining lifespan in worms, flies, and mice) should also be considered in light of the impact of this signaling pathway on dementia (cognition) and neurogenesis.

Beyond regulating glucose metabolism, insulin and IGF-1 control neuronal and glial cell survival, synapse function, memory, cognition, and anti-inflammatory actions ^61^, with evidence for insulin resistance contributing to both the development and the progression of AD ^62^. AD is characterized by amyloid beta (Aβ) plaques, phospho-tau protein (p-tau) neurofibrillary tangles, cortical neuron loss, and cognitive impairment ^63, 64^. Aβ plaques and pTau are the defining characteristics of AD, with glycogen synthase kinase 3β (GSK3β) representing Tau Kinase 1 and the source of an alternative hypothesis of AD in which GSK3β overactivity leads to AD ^65, 66^. It has been hypothesized that CNS insulin resistance may play a causal role in both the development and the progression of AD, consistent with insulin’s role in brain metabolism and neuronal survival ^61^. Of note, insulin and IGF-1 resistance and deficiency are typically observed early in the progression of AD and the abnormal molecular and biochemical alterations parallel those seen in type 1 diabetes mellitus (T1DM)/T2DM, leading some to use the term “type 3 diabetes mellitus” (T3DM) to reflect the concept that insulin resistance within the brain may cause AD ^50, 61, 67– 69^. A caveat of these studies is that much of what is known about IGF-1/insulin signaling in the CNS is based on studies with rodents; this has been a source of concern with respect to the translation of these findings to humans ^16^. A recent review by Arnold and co-workers details the overlap between patients with AD and T2DM, brain dysfunction in T2DM, and the fact that brain insulin resistance occurring in T2DM may lead to cognitive impairment ^70^.

In a recent study examining the relationship between AD and IGFBP2 levels across a spectrum of 300 human AD patients and two transgenic mouse models of AD, investigators found cerebrospinal fluid (CSF) IGFBP2 levels correlated with CSF tau levels and brain atrophy in non-hippocampal regions ^71^. This study linked IGFBP2 levels to tau pathology, which may, in turn, reflect a decrease in IGF-1 signaling resulting from IGF-1 sequestration by IGFBP2. This is consistent with an early observation of localized diabetes within the brain, which noted that, in the absence of systemic symptoms of diabetes, insulin and IGF-1 resistance were detectable in the hippocampus and, to a lesser extent, the cerebellar cortex ^41– 43^. These measurements were based upon observations of reduced responsiveness to insulin in the IR/IRS1/PI3K cascade and to IGF-1 in the IGF1R/IRS2/PI3K pathway. Reduced signaling through both IRS1 and IRS2 in this context has consistently revealed elevated serine phosphorylation of IRS1 (pS ^616^, pS ^636/639^/IRS2), mediated by cJun N-terminal kinase (JNK)1/2, extracellular signal regulated kinase (ERK)1/2, mammalian target of rapamycin (mTOR), AKT, and GSK3β ^41– 43, 68^.

## T2DM is a risk factor for AD

AD is a degenerative metabolic disease caused by brain insulin resistance and deficiency and overlapping with the molecular, biochemical, pathophysiological, and metabolic dysfunction in diabetes mellitus ^61^. In this context, insulin, IGF-1, incretins, and insulin sensitizers may be useful for treating different stages of neurodegeneration. Both diseases have a protein-misfolding component, and patients with T2DM have an increased risk for AD ^72^. Streptozotocin (STZ) is a glucosamine-nitrosourea diabetogenic agent used to generate T1DM in rodents based on its preferential uptake into pancreatic beta cells via Glut2 transporters, nuclear entry, alkylation of DNA, and beta cell death ^73^. At low doses, STZ can induce T2DM and insulin resistance in the CNS following intracerebroventricular administration ^74, 75^. Insulin and IGF-1 resistance in human T2DM significantly increases the risk of AD ^71, 76^. Over the last several years, the increased incidence of AD and T2DM in the aging population has emerged. Of note, ineffective insulin/IGF-1 signaling resulting from insulin resistance is a risk factor for AD, where the presence of T2DM doubles the risk of AD ^16, 61, 76^. Liraglutide, an insulin secretagogue, and other drugs available for treating T2DM are strong candidates to repurpose for AD and hit multiple targets, representing an example of polypharmacology over polypharmacy in the repurposing of drugs for their side effects in AD ^77^. In addition, clinical trials testing the efficacy of intranasal administration of long-acting detemir insulin showed that this form of insulin gets into the brain with reduced associated systemic hypoglycemic episodes and mild cognitive improvement ^78^. Delivery of intranasal insulin in humans was found to increase dorsal medial PFC–hippocampal functional connectivity with the potential to improve cognition and metabolism ^79^. Of note, intranasal administration of insulin in wild-type (WT) mice versus a mouse model of AD (APP _Swe_/PS1 _dE9_ [APP/PS1] transgenic mice) was effective only in the WT mice ^80^. The insulin sensitizer metformin is currently being evaluated for use in patients with mild cognitive impairment in a national clinical trial (“Metformin in Alzheimer’s Dementia Prevention [MAP]”; NCT04098666).

## Conclusion and future perspectives

As with the ongoing controversy of whether the IGFBPs possess biologic functions independent of the binding and sequestration of the IGFs to directly inhibit or stimulate tumor growth, a similar disagreement in the context of whether centrally acting IGF-1/insulin stimulates or inhibits cognition/neurogenesis/dementia exists. As additional human studies and clinical trials test the efficacy of insulin/IGF-1 in modulating cognition along with the refinement of their more selective targeting to brain regions like the hippocampus over the hypothalamus, more desirable outcomes may emerge. With respect to the cross-talk between cancer and AD, a recent population-based study showed that patients with a history of cancer had a lower incidence of AD ^81^. It was further noted that older individuals with cancer had better memory and slower memory decline, supporting the hypothesis that there may be a common pathologic process working in an opposing manner in cancer and AD. This study lends further support to this previously identified inverse relationship that has been described by others ^82, 83^. Though we currently lack a mechanism underlying this phenomenon, it is tempting to speculate that IGF-1 and insulin/IGF-1 signaling could be the common denominator.

## Abbreviations

Aβ, amyloid β peptide; AD, Alzheimer’s disease; Akt, Ak strain transforming; protein kinase B; Bim, Bcl-2-interacting mediator of cell death; CSF, cerebrospinal fluid; CNS, central nervous system; CUL7, Cullin 7; DILP, *Drosophila* insulin-like peptide; DKO, double knockout; FOXO3A, forkhead box O3A; GSK3β, glycogen synthase kinase 3β; HIT, hepatic IGF-1 transgenic; IBP, insulin-like polypeptide-binding protein; IGF, insulin-like growth factor; IGFBP, insulin-like growth factor binding protein; IGF1R, insulin-like growth factor 1 receptor; IGF2R, insulin-like growth factor 2 receptor; Imp-L2, imaginal morphogenesis protein-late 2 protein; IR, insulin receptor; IRS, insulin receptor substrate; RTK, receptor tyrosine kinase; mAb, monoclonal antibody; NSCLC, non-small cell lung cancer; PI3K, phosphoinositide 3-kinase; T1DM, type 1 diabetes mellitus; T2DM, type 2 diabetes mellitus; Tyr, tyrosine; STZ, streptozotocin; WT, wild type.
